# Gene expression profiling in juvenile and mature cuttings of *Eucalyptus grandis* reveals the importance of microtubule remodeling during adventitious root formation

**DOI:** 10.1186/1471-2164-15-826

**Published:** 2014-09-30

**Authors:** Mohamad Abu-Abied, David Szwerdszarf, Inna Mordehaev, Yossi Yaniv, Saar Levinkron, Mor Rubinstein, Joseph Riov, Ron Ophir, Einat Sadot

**Affiliations:** The Institute of Plant Sciences, Volcani Center, PO Box 6, Bet-Dagan, 5025000 Israel; The Robert H. Smith Institute of Plant Sciences and Genetics in Agriculture, The Robert H. Smith Faculty of Agriculture, Food and Environment, The Hebrew University of Jerusalem, Rehovot, 7610001 Israel; Syngenta Seeds – R&D, Valle de Azapa Km 17, Arica, Chile

**Keywords:** *Eucalyptus grandis*, Juvenile-to-mature phase change, Adventitious-roots formation, Microarray analysis, Microtubule

## Abstract

**Background:**

The ability to form adventitious roots (AR) is an economically important trait that is lost during the juvenile-to-mature phase change in woody plants. Auxin treatment, which generally promotes rooting in juvenile cuttings, is often ineffective when applied to mature cuttings. The molecular basis for this phenomenon in *Eucalyptus grandis* was addressed here.

**Results:**

A comprehensive microarray analysis was performed in order to compare gene-expression profiles in juvenile and mature cuttings of *E. grandis,* with or without auxin treatment on days, 0, 1, 3, 6, 9 and 12 post AR induction. Under these conditions AR primordia were formed only in auxin-treated juvenile cuttings. However, clustering the expression profiles revealed that the time after induction contributed more significantly to the differences in expression than the developmental phase of the cuttings or auxin treatment. Most detected differences which were related to the developmental phase and auxin treatment occurred on day 6, which correlated with the kinetics of AR-primordia formation. Among the functional groups of transcripts that differed between juvenile and mature cuttings was that of microtubules (MT). The expression of 42 transcripts annotated as coding for tubulin, MT-associated proteins and kinesin motor proteins was validated in the same RNA samples. The results suggest a coordinated developmental and auxin dependent regulation of several MT-related transcripts in these cuttings. To determine the relevance of MT remodeling to AR formation, MTs were subjected to subtle perturbations by trifluralin, a MT disrupting drug, applied during auxin induction. Juvenile cuttings were not affected by the treatment, but rooting of mature cuttings increased from 10 to more than 40 percent.

**Conclusions:**

The data suggest that juvenile-specific MT remodeling is involved in AR formation in *E. grandis*.

**Electronic supplementary material:**

The online version of this article (doi:10.1186/1471-2164-15-826) contains supplementary material, which is available to authorized users.

## Background

Rooting capability is one of the economically important traits that are lost during the juvenile-to-mature phase change in woody plants. The difficulties in propagation of promising clones of woody plants, such as rootstocks of fruit trees, ornamental woody plants, and forest trees, hampers breeding programs that depend on the production of rooted cuttings.

The process of maturation in plants is characterized by progressive changes in various morphological and developmental traits, among which is ARs formation [1–5]. The following four phases have been defined in plant development: (i) embryonic phase, (ii) postembryonic juvenile vegetative phase, (iii) mature vegetative phase, and (iv) mature reproductive phase [5, 6]. In woody plants, the juvenile traits, including rooting capability, are expressed at the base of the stem throughout the life of the plant and are sharply or gradually replaced by maturity traits, including rooting inability, toward the upper part of the main stem and branches [5].

AR formation is a complex process, in which roots differentiate and regenerate from non-root tissues [7, 8], and is often described to occur in four steps: (i) cell de-differentiation, (ii) cell division, (iii) development of root primordia, and (iv) root emergence. Auxin plays a major role in each of these steps [9, 10]. Histological analysis of woody plants induced to form AR revealed that cell division (step ii) is induced in both juvenile and mature tissues, but differentiation of root primordia (step iii) occurs efficiently in juvenile cuttings but is compromised in mature cuttings [11–13]. Mature cuttings have often been reported to produce callus tissue instead of root primordia.

Data have been accumulated using DNA-chip analysis regarding the molecular regulation of AR formation in woody plants. In poplar (*Populus tremula x P. alba*), the expression of genes related to ethylene-biosynthesis pathway, auxin-response factors (ARF), IAA family members, and cytokinin-regulated transcripts was reported to change during the first 48 h following root induction [14]. The expression of few genes was modified in transgenic poplar plants overexpressing the cytokinin-response regulator *PtRR13*, in which AR formation was inhibited [14]. These included the gene encoding PLEIOTROPIC DRUG RESISTANCE TRANSPORTER 9 (PDR9), which is involved in auxin efflux, the genes encoding FIMBRIN-LIKE2 (FIM2), an actin-binding protein and CONTINUOUS VASCULAR RING1, and two genes encoding APETALA2/ ETHYLENE RESPONSE FACTOR [14]. Chip analysis of RNA samples collected 3–4 and 5–6 days after AR induction in cuttings of the poplar hybrids *P. tremula x P. tremuloides* (T89) and *P. tremula x P. alba* (717-1B4), revealed that *AINTEGUMENTA LIKE1,* a transcription factor of the *AP2* family, is also involved in AR formation [15]. Using activation tagging in the *Populus* clone 717-1B4, another gene from the AP2/ERF family, *PtaERF003*, was found to promote AR formation [16]. Recently, WUSCHEL (WUS)-related homeobox (WOX) protein family members were shown to promote AR formation in transgenic hybrid poplar [17].

In *Pinus contorta*, gene-expression profiles were determined at several time points from day 0 till 33 days after root induction. Genes involved in cell division cell-wall weakening, and those related to water stress were upregulated during the initial stages. During later stages, genes involved in cell replication and stress were downregulated, suggesting maturation and functioning of the new roots. During the phase of root-meristem formation, the expression levels of genes involved in auxin transport and auxin-responsive transcription increased [18]. Gene-expression analysis performed in juvenile rooting-competent pine (*Pinus radiata*) and chestnut (*Castanea sativa*) cuttings revealed the induction of *SCARECROW-LIKE (SCL)* genes 24 h after auxin application [19], and the specific expression of *CsSCL1* in the cambium and adjacent cells [20]. In *P. radiata*, a (short root) *SHR*-like gene was expressed in the cambial region of rooting-competent cells of hypocotyl cuttings within the first 24 h after the initiation of rooting and before the activation of cell division [21].

AR formation has been studied in *Eucalyptus* species [22–24], and analysis of the transcriptomes of juvenile and mature *E. grandis* cuttings prior to root induction revealed numerous differences in gene expression patterns [25]. One of the transcripts that was upregulated in juvenile rooting-competent cuttings was *EgNIA* (nitrate reductase), which is known to regulate nitric oxide (NO) production, which in turn promoted AR formation [25]. In addition, by profiling micro RNAs in *E. grandis* we have found that there was no mutual correlation between the expression of miR156 or miR172 and rooting potential or loss of rooting potential respectively [26]. In the present study we determined the kinetics of AR formation and performed a comprehensive analysis of transcriptional profiles in order to compare auxin responsiveness in juvenile and mature cutting of *E. grandis* during 12 days of AR induction.

## Results

### The kinetics of root primordia formation in *E. grandis*cuttings

**Figure 1:**
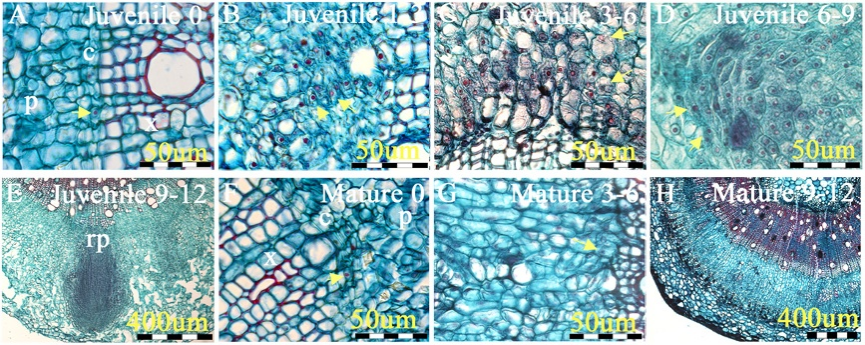
**Histological analysis of the kinetics of AR formation in juvenile and mature cuttings of** ***E. grandis*** **.** Cuttings were treated with IBA before placing in rooting tables. The bottom 1 cm of each cutting was fixed, embedded in paraffin and sectioned. **A-E** Juvenile cuttings. **F-H** Mature cuttings. Numbers refer to days after excision. Yellow arrows show meristematic cells. Phloem-p, Xylem-x, Cambium-c, Root promordia-rp.

The present study was undertaken to elucidate the transcriptional differences between juvenile and mature cuttings following excision, exogenous application of auxin and during incubation of cuttings in rooting tables until AR are formed. To determine the kinetics of root-primordia formation, cuttings were processed for histological analysis immediately after excision and 1, 3, 6, 9 and 12 days after induction with auxin (Figure 1). Auxin-treated juvenile cuttings exhibited cell division near the cambium cells, located between the phloem and xylem layers, 1–3 days after induction (Figure 1B). At 3–6 days, dome-like primordia started to appear (Figure 1C), and continued to develop during 6–9 days (Figure 1D). At 9–12 days, elongated and well-developed AR primordia were observed (Figure 1E), and at the end of this period, roots started to emerge. In contrast, in mature cuttings, although meristematic cells were detectable adjacent to the cambium (Figure 1F) on the day of excision, and small clusters of them were detected after 3 to 6 days (Figure 1G), almost no differentiated primordia were detected after 9 to 12 days (Figure 1H and [25, 26]). These observations are in agreement with previous reports which showed cell division but no root differentiation in auxin treated mature tissues of various tree species [11–13].

### Transcriptome analysis during AR formation

**Figure 2:**
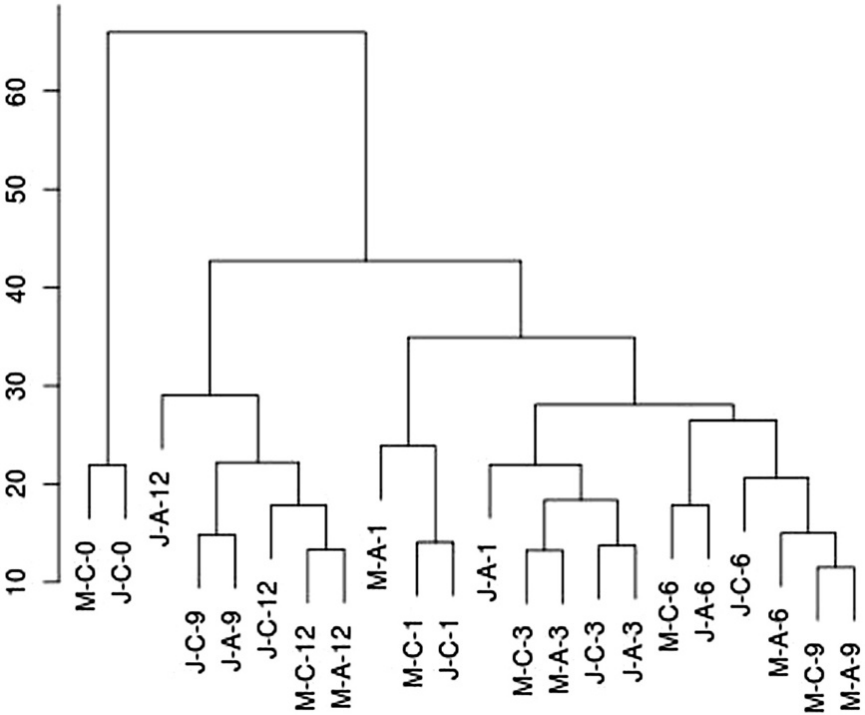
**Clustering of the different expression profiles.** RNA from control juvenile (J-C), auxin-treated juvenile (J-A), control mature (M-C) or auxin-treated mature (M-A) cuttings, was extracted immediatly (0) and 1, 3, 6, 9 and 12 days after excision and hybridized to a DNA chip containing 15,744 transcripts. The results were clustered as described in methods.

**Figure 3:**
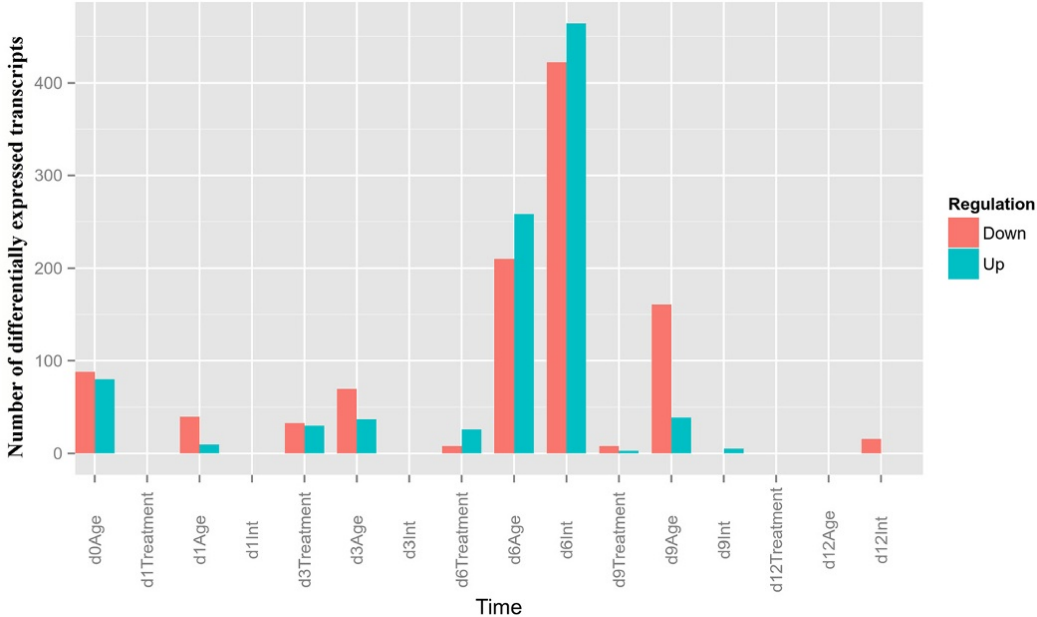
**Distribution of differentially expressed transcripts.** The contribution of developmental phase (Age), auxin (Treatment), and their interaction (Int) is presented here as the number of statistically significant (p-value < 0.05) up-regulated (fold change > 2; blue) and down-regulated (fold change < 0.5; red) genes. Differential expression was calculated by applying LIMMA R-package statistical approach (see methods section for details) in each time point (day 1, 3, 6, 9, and 12) after excision. An exception is the day of excision (d0) in which only the effect of developmental phase (age) was detected.

To shed light on the transcriptional differences between juvenile and mature cuttings, we designed an experiment in which these cuttings were either treated or not treated with auxin and incubated on rooting tables. This enabled distinguishing between transcripts affected by auxin and those affected solely by the wounding and incubation. It should be noted that after 12 days, root primordia appeared only in auxin treated juvenile cuttings, but not in untreated ones or in treated or untreated mature cuttings. RNA was extracted from three biological replications on the day of excision (0) and 1, 3, 6, 9, and 12 days after induction (total of 66 samples) and was subjected to comprehensive DNA-microarray analysis. Clustering the expression profiles showed that the time after excision was the major contributor to the differences in expression (Figure 2). Thus, most transcripts of juvenile and mature cuttings, which were incubated on the rooting table for the same period of time clustered together regardless of auxin treatment. This suggests that the expression of root specific transcripts in the auxin treated juvenile cuttings after 12 days is not high enough to distinguish them from the non rooting cuttings when determined by clustering analysis. The distribution of upregulated and downregulated transcripts (twofold; adjusted *P*-value < 0.05) over time after excision revealed the following trends (Figure 3). Auxin treatment began to have a detectable effect only after 3 days, which remained relatively low for 6 and 9 days and disappeared after 12 days. Age (developmental phase) had a stronger effect, as reflected by the large number of transcripts that were down- or upregulated in juvenile cuttings compared to mature ones. On day 6, the number of differentially expressed transcripts between juvenile and mature cuttings and between auxin-treated and -untreated was the largest. In addition, there was a clear increase in the effect of the interaction between auxin treatment and age on day 6 (Figure 3). This is an indication that a critical transcription difference between juvenile and mature *E. grandis* tissues occur at this time window during AR induction. Out of the 15,744 probes printed on the chip, a total of 1,790 transcripts showed significant changes (twofold; adjusted *P*-value < 0.05) in expression between juvenile and mature cuttings (Figure 3). These transcripts included a group of 40 transcription factors (2.2%), among them putative family members of MYB-, WRKY- and NAC-domain-containing proteins, SCL, and the auxin-related transcription factors, IAA and AUX. In addition, 63 protein kinases (3.5%), among them transcripts encoding putative leucine-rich-repeat transmembrane protein kinases, calmodulin-dependent protein kinase, shikimate kinase family protein, casein kinase-like, phosphatidylinositol-4-phosphate 5-kinase family protein, and SOMATIC EMBRYOGENESIS RECEPTOR-LIKE KINASE 1; a smaller group of redox enzymes, among them oxidases, peroxidases, and reductase; cell wall enzymes, among them cellulose synthase subunits, pectinesterases, polygalacturonase, expansin-like, and arabinogalactans; auxin- and gibberellin-related transcripts, among them PIN3- and PIN4-related proteins, ARF6- and ARF8-related proteins, auxin-responsive proteins, gibberellin-responsive proteins, and transcripts involved in gibberellin metabolism, GA20OX, and GA2OX; microtubule (MT)-associated proteins, among them several kinesins and MAP70; actin- and membrane-trafficking-related proteins, among them exocyst complex subunits, clathrin heavy chain, Rab proteins, myosins and other actin-binding proteins (for all the data please see GEO accession number GSE57375). The data suggest that multiple transcripts related to different regulatory processes differ in their expression between juvenile and mature cuttings during AR induction, including those that are involved in post-translational modifications, such as phosphorylation, remodeling of the cell wall and the cytoskeleton, and changes in membrane trafficking.

**Figure 4:**
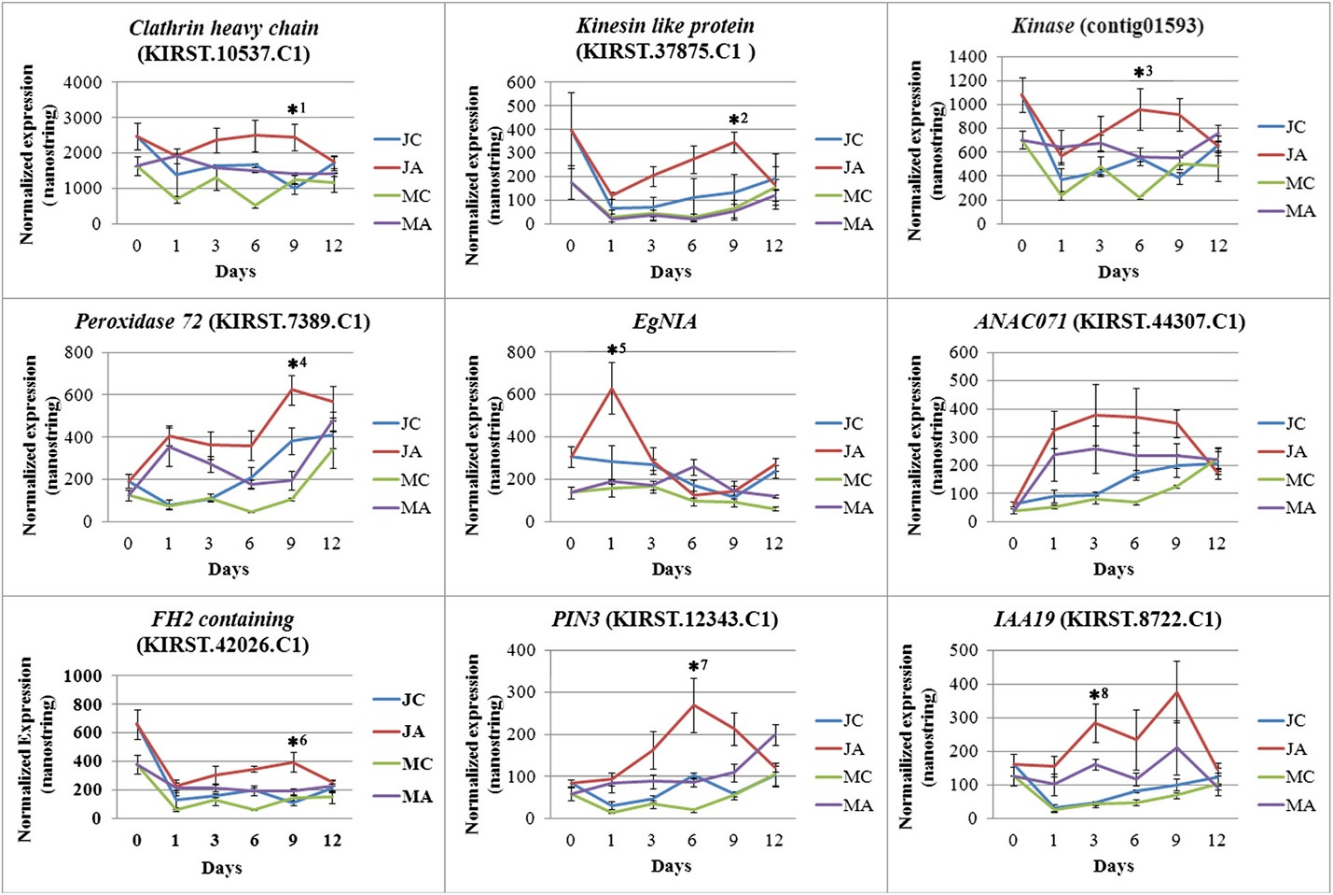
**Validation of expression of several transcripts by the Nanostring method confirms differential response of some transcripts to auxin in juvenile and mature cuttings.** The same RNA samples which were used for the chip analysis were used for the validation. Probes were designed for some selected transcripts and a nanostring detection was performed. Asteriks show statistical significant difference determined by Scheffe analysis as follows: *^1^: JA vs JC p < 0.05, *^2^: JA vs JC p < 0.05, JA vs MC and MA p < 0.01, *^3^: JA vs MA p < 0.05, JA vs MC p < 0.01, *^4^: JA vs MC and MA p < 0.01, *^5^: JA vs MC and MA p < 0.05, *^6^: JA vs JC and MA p < 0.05, JA vs MC p < 0.01, *^7^: JA vs MC and MA p < 0.01, JA vs JC P < 0.05, *^8^: JA vs JC and MC P < 0.05.

Although the contribution of auxin to the total differential expression was low (Figures 2 and 3), some transcripts from each functional group showed a differential auxin responsiveness between juvenile and mature cuttings. The expression of some of these transcripts was validated by the Nanostring method [27]. Figure 4 shows that the expression of *E. grandis* homologs of clathrin heavy chain, kinesin-like protein, a kinase like, peroxidase 72, NIA (nitrate reductase), a transcription factor containing a NAC domain, PIN3, IAA19, and an FH2 containing protein was higher at a certain time point, in auxin treated juvenile cuttings compared to mature ones.

### The MT system is differentially regulated in juvenile and mature cuttings

**Figure 5:**
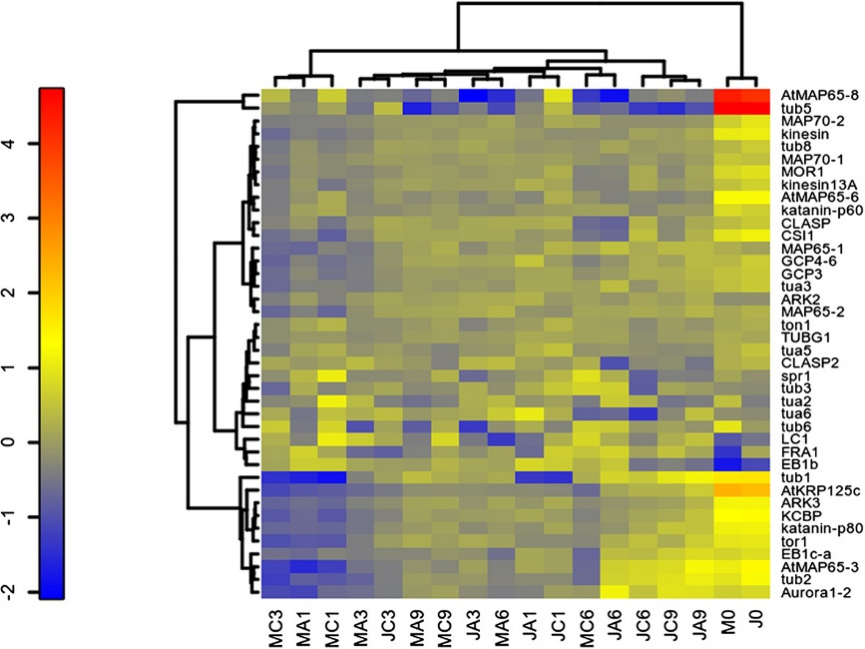
**Analysis of expression of microtubule-related transcripts during induction of AR formation.** MC, mature, control; MA, mature, auxin-treated; JC, juvenile, control; JA, juvenile, auxin-treated. Samples from days 0, 1, 3, 6 and 9 after excision. The same RNA samples used for the microarray analysis were used for the validation using the nanostring method. Clustering was done as described in methods.

One of the functional groups whose members showed expression differences between juvenile and mature cuttings was that encoding MT-associated proteins (MAPs). We therefore hypothesized that unlike the clusters shown in Figure 2, clustering expression profiles based on MAP expression might lead to segregation between juvenile and mature samples. In addition, MTs play essential regulatory roles in cell division [28, 29] and cell elongation [30–32], and a recently presented evidence suggests that they have additional functions in programs related to organogenesis [33]. Therefore, we searched a unified EST database [26], and the recently released genome sequence [34] for transcripts related to MTs. The list (Additional file 1: Table S1) includes: homologs of 9 *TUBULIN* β, 5 *TUBULIN* α, 6 *MAP65*, 2 *MAP70*, 2 *CLASP*, *KINESIN 13A, armadillo-repeat-containing kinesins ARK2 and ARK3, KINESIN-LIKE CALMODULIN-BINDING PROTEIN (KCBP), KRP125c, PAK kinesin-like*, 2 *EB1*, *GCP3/SPC98*, *GCP4/6*, γ *TUBULIN*, *MOR1*, outer *DYNEIN*-like light chain, *KATANIN p60*, *KATANIN p80*, *TOR1*, *SPR1*, *TON1*, *AURORA1/2*, and *CSI1*. Although this might not be the full list of MT-associated proteins in the *Eucalyptus* genome, their expression profiles during root induction in juvenile and mature cuttings should indicate whether there are major differences in MT remodeling between these two types of cuttings. Probes were designed (Additional file 1: Table S1) and expression levels were determined by the Nanostring method in RNA samples similar to those used for the microarray analysis (Figure 5). The probes designed for the following homologs: *FRA1*, *DYNEIN*-like light chain, *SPR1*, *TUBILIN* α2 and α6, *TUBULIN* β3 and β6, and for one of the *CLASP* predicted transcripts, gave signals that were very close to the background signal, and therefore these transcripts might be expressed at very low levels or represent pseudo genes. The *E. grandis* homologs of *AURORA1/2*, *TUBULIN β2*, *MAP65-3*, and *EB1c/a*, clustered together (Figure 5) and their expression was significantly higher in the juvenile samples compared to the mature samples on days 6 and 9 (Additional file 2: Figure S1). In addition, in contrast to the cluster shown in Figure 2, which was based on the expression of more than 15,000 transcripts, here the profile of juvenile cuttings, 6 and 9 days after excision, with or without auxin treatment, clustered in close proximity. These results indicate that differential remodeling of MT occurs in juvenile cuttings compared to mature cuttings and might be relevant to the shift from cell division to cell differentiation during formation of AR primordia. Of note, MT remodeling might as well be the result and not the cause of cell differentiation.

### Subtle perturbations of MTs improve rooting of mature cuttings

**Figure 6:**
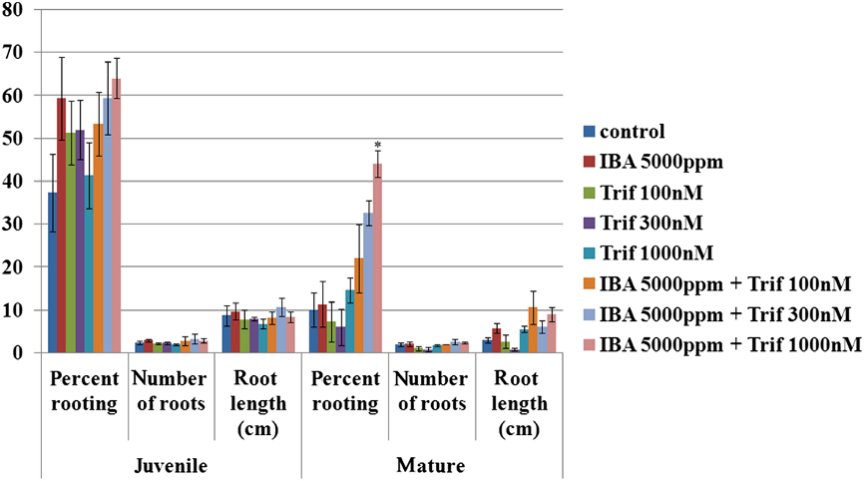
**Induction of AR formation in juvenile and mature cuttings in the presence of the MT disrupting drug trifluralin.** Juvenile and mature cuttings were treated with 5000 g/liter K-IBA and/or increasing concentrations of trifluralin for 20 sec. Rooting was scores after 30 days. Bars represent averages ± SE, asterik show statisticaly significant difference as determined by Scheffe analysis p < 0.05.

As mentioned above, during rooting of woody plants mature cuttings, callus is often formed instead of roots. This phenomenon occurs because cell division is activated whereas cell differentiation is inhibited. We hypothesized that if MTs play a role in differentiation, and their dynamics in mature cuttings does not favor differentiation, then subtle perturbations might make a difference. Juvenile and mature cuttings were treated with auxin and increasing concentrations of the MT-disrupting drug trifluralin (Figure 6). While juvenile cuttings were not affected by trifluralin, rooting rate of mature cuttings gradually increased from 10 to 40 percent, concomitant with the increase in trifluralin concentration.

## Discussion

The loss of rooting ability in mature woody plants has mystified plant scientists and growers for years. *E. grandis* exemplifies this problem. Pruning of 6- to 7-month-old *E. grandis* seedlings at either 10–15 cm or 150–200 cm above the ground induces shoot sprouting near the cut trunk. Cuttings taken from low-pruned seedlings root easily, whereas cuttings taken from high-pruned seedlings barely root at all [24–26]. This phenomenon demonstrates the gradual decrease of rooting ability in physiological mature tissues. However, the specific maturity traits that inhibit rooting are unknown. Completion of sequencing of the *E. grandis* genome [34] has made it a suitable experimental system for addressing these questions. In the present study we analyzed the gradual changes in expression profiles of the expression of 15,000 transcripts during the root-induction process in juvenile and mature cuttings in the presence or absence of auxin. This enabled us to distinguish between expression changes mediated by the developmental stage of the cuttings, and those mediated by the auxin treatment. Selection of the time intervals at which RNA was extracted during root induction was based on the kinetics of root primordia formation. In juvenile cuttings, clusters of dividing cells were already detectable after 1–3 days, root primodia were formed after 6–9 days, and root emergence was detectable after 12 days in auxin-treated juvenile cuttings. In contrast, in mature cuttings, although some cell divisions were observed, no primordia were formed during the time period of the experiment. This is in agreement with observations made in other tree species [11–13], and suggests that the transition from cell division to cell differentiation is somehow hampered in mature cuttings.

It was found here that the most significant expression differences were driven by the time after excision, less by the developmental phase of the cuttings, and the least by auxin treatment. This is in agreement with previous observations in pine, in which no differences in auxin uptake, metabolism and transport were found between easy-to-root hypocotyls and difficult-to-root epicotyls [35]. It should be emphasized that rooting was not synchronized in all cuttings, and that the frequency of root primordia in the samples taken for RNA preparation was low. Therefore, the results may be masked by a high background of irrelevant transcripts expressed by stem tissues surrounding the primordia. This is an intrinsic problem of the biological system, and we cannot rule out the possibility that important transcripts went undected. Despite this problem some statistically significant expression differences were revealed by the system. These offer some novel research directions for AR-formation research. For example, the finding that 3.5% of the transcripts that were differentially expressed between juvenile and mature cuttings are protein kinases, suggests that major differences are mediated by post-translational modifications, such as phosphorylation, protein–protein interactions, and protein stability. Indeed, receptor-like kinases play an important role in root development [36].

The finding of a transient increase in NIA after 1 day of auxin treatment only in juvenile cuttings is in agreement with our previous data, which showed that *E. grandis* NIA is involved in a transient increase in NO concentration after excision [25]. We also showed that higher levels of NO accumulates in juvenile cuttings than in mature ones after pruning [25], which is likely due to the observed specific auxin-mediated NIA upregulation in these cuttings. The difference in expression of clathrin and the formin-like FH2 domain containing transcript between juvenile and mature cuttings, suggest differences in remodeling of the actin and membrane trafficking systems during AR induction. This is in agreement with previous observations which showed differential expression of actin in response to IBA between pine hypocotyls and epicotyls [37], of actin7 between juvenile and mature cuttings of *Eucalyptus grandis* [25], and a change in a FIMBRIN-LIKE2 (FIM2) in transgenic poplar with inhibited AR formation potential[14]. The relevance of the actin system to AR formation was also suggested when paradoxically, a RGD containing peptide improved rooting of mature *Arabidopsis* cuttings [38].

The changes in expression of MT-related transcripts suggest that they are also involved in AR organogenesis. The notion of MT involvement in plant organogenesis has recently been addressed and a cross talk between MT and auxin signaling has been found [33]. We presume that MTs participate in the regulation of four factors that are important for AR formation: (1) the number of dividing cells, which should reach a threshold level for coordinated differentiation to occur; (2) the orientation and symmetry of cell division; (3) the cross talk and coordination between dividing cells which allows organogenesis; and (4) polar cell elongation. The largest difference in gene expression after 6 days might reflect a peak in the extent of cell division in juvenile cuttings at this time. This assumption is based on three different observations: (1) the histological analysis that shows much more dividing cells in juvenile cuttings than in mature ones. (2) the specific increase in Aurora, and MAP65-3 in juvenile cuttings compared to mature ones. Both proteins participate in the regulation of the MT apparatus during cell division [39, 40]; (3) toward day 12 the differences in expression profiles between juvenile and mature cuttings decline despite root primordia formation, suggesting that root-specific genes do not underlie the major expression differences detected in this system. It might be concluded that the shift to differentiation depends among other things on a critical mass of dividing cells, below of which differentiation is hampered. In addition, asymmetric cell division which is also regulated by MTs [41] is critical for differentiation [42]. Based on our finding we propose that differential permissive conditions which exist in the context of juvenile tissues in contrast to mature tissues and are influenced by MT remodeling allow the shift from cell division to cell differentiation after the initial onset of cell division.

The increase in rooting rate after subtle perturbation of the MTs with trifluralin might result from changes in MT dynamics, organization, or mass which affect cell division [28, 29], cell-wall properties [43], coherent auxin transport [44], or other unknown parameters. Of note, the half life of trifluralin was documented to be 25–201 days under various agronomic conditions [45] which suggests that its effect may last throughout AR induction.

## Conclusions

Taken together, our data indicate that the difference in expression profiles between juvenile and mature *E. grandis* cuttings is mostly affected by time after excision, to a lesser extent by the developmental stage of the cuttings, and least by auxin treatment. Among the functional groups of genes that were differentially expressed during AR induction in these cuttings, was that of the MAPs. Therefore, juvenile-specific, fine-tuned MT remodeling seems to be involved in AR formation. Induction of juvenile specific expression that is observed 6–9 days after excision and auxin application is thought to be a second or a third wave of transcription activation that is induced by early genes that are induced shortly after the treatment. Such immediate induction was previously described for *SHORT-ROOT like* gene from *Pinus radiata* [21] and *SCARECROW-like* genes from *Pinus radiata* and European chestnut *Castanea sativa* Mill in cambium cells of competent cuttings [19, 20]. We propose that both early and late changes in gene expression are giving the tissue its rooting competence. Nevertheless, some expression patterns observed several days after root induction might also be the result of root differentiation and not the cause for it.

## Methods

### Plant material

Seeds of *E. grandis* were grown for 1 month after germination in pots and then transplanted into 15-l pots containing peat and tuff (70:30) and 2 g/l Osmokot. Six-month-old seedlings grown in a net-house were pruned at 10–15 cm and 150–200 cm above the ground to enhance cutting production along the pruned stems (juvenile and mature, respectively). Cuttings consisting of 4–6 leaves with 60% of the leaf blade removed were used. After excision, the base of the cuttings was dipped for 20 s in a solution containing 6 g/l K-IBA (Sigma). Cuttings which were not treated with auxin were used as control. Both treated and untreated cuttings were incubated on a rooting table heated to 24°C under 90% relative humidity for a period of 14–42 days [25, 26]. For trifluralin (32061 FLUKA) treatment, cuttings were treated with 100, 300, or 1000 nM trifluralin with or without 6 g/l K-IBA for 20 sec.

### Histological analysis

Juvenile and mature cuttings were induced to root with 6 g/l K-IBA as described above. Samples were taken at 0, 3, 6, 9 and 12 days after excision. Tissues were fixed in FAA (50% ethanol, 5% glacial acetic and 4% formaldehyde) overnight at room temperature. Tissues were gradually dehydrated in ethanol series (75%, 90% and 100%) for 1 h each, and then the ethanol was gradually replaced with histoclear (Gadot) in five steps of 1:3, 1:1, 3:1 and two steps of pure histoclear (1 h each). The histoclear was then gradually replaced with paraffin (PARAPLAST X-TRA, Leica). Sections (15 μm) were made with rotary microtome (Leica RM2255) and stained with Safranin and fast-green.

### RNA isolation

RNA extraction was carried out as previously described [46], with some modifications. Tissue (3 g) was ground in liquid nitrogen to a fine powder using a mortar and pestle, and then 10 ml of extraction buffer preheated to 65°C was added, and the tubes were shaken thoroughly and incubated for 5 min at 65°C. The extraction buffer contained 2% cetyltrimethylammonium bromide (CTAB), 2% polyvinylpyrrolidone (PVP) 40, 100 mM Tris–HCl pH 8.0, 25 mM EDTA, 2 M NaCl, 0.5 g/l spermidine, and 2% β-mercaptoethanol. All reagents were freshly prepared. Two steps of chloroform extraction were performed, with centrifugation at 10,000 *g* for 15 min after each step. The upper phase was mixed with 0.25 volume of 10 M LiCl, and the RNA was precipitated overnight at 4°C. Following centrifugation at 10,000 *g* for 20 min, the RNA pellet was dissolved in 500 μl SSTE buffer containing 0.5% SDS, 1 M NaCl, 10 mM Tris–HCl, pH 8.0, and 1 mM EDTA, and then extracted with an equal volume of chloroform. The RNA was then precipitated by the addition of 2 volumes of ethanol held at -70°C for 1 h, and then pelleted by centrifugation at 10,000 *g* in a microcentrifuge at 4°C. The pellet was dried and then dissolved in RNAse-free water. The RNA sample was further purified with the Qiagen RNeasy mini kit and treated with RNase free DNase I (Qiagen).

### Chip design and analysis

An Agilent array of 18 K probes was designed based on the eucalyptus version 1 array of 44 K probes. A detailed description of probes selection is provided in our previous study [25]. In version 2, a subset of 18 K probes was taken, with first priority given to the probes that were found statistically significant in version 1 [25]. Version 2 accession at GEO NCBI is GSE57375.

### Statistical analysis

Three replicas of RNA samples from each juvenile or mature cuttings, either treated with auxin or not, 0, 1, 3, 6, 9, or 12 days after excision (total of 66) were hybridized to our custom-made Agilent array. Initially, loess and Aquantile normalization was performed, followed by calculating a moderated Student’s t-test using the **Li**near **m**odels for **m**icro**a**rray (Limma) package [47]. This t-statistic allows for better variance estimation by using an empirical Bayesian approach. The probes' log signal ratios were ranked by their adjusted *P*-values (*q-*values), and were then selected for genes with significantly different expression (*q* < 0.05). The correction for multiple comparisons was performed using Benjamini and Hochberg’s false discovery rate (FDR) [48]. The analysis was performed for each time point separately in a loop factorial design for control, juvenile, mature, and IBA-treatment.

Hierarchical clustering was run by first converting the dual-channel results into single channel as described in the separate channel analysis section in the Limma user's guide. The next agglomerative hierarchical algorithm was applied with “ward” method parameter.

### Transcript-expression analysis

Expression analysis of selected transcripts was performed by Nanostring (http://www.Nanostring.com). The probes used are listed in Additional file 1: Table S1. Two-way clustering of Nanostring expression signals was performed after normalization of the gene signals to positive controls and to a housekeeping gene (ubiquitin, Contig11492) embedded in the Nanostring platform. Scaling of the gene expression signals was performed by subtracting the mean of the log_2_ signals. An agglomerative hierarchical algorithm was applied with the “ward” method on both the genes and the samples, and a heatmap was generated by “Heatplus” R-package.

## Electronic supplementary material

Additional file 1: Table S1: Primers used for the Nanostring analysis. (XLSX 16 KB)

Additional file 2: Figure S1: Scheffe analysis of the expression of four microtubule related transcripts. Chart represent averages and standard errors, and asteriks show statisticaly significant differences. Expression was determined by the nanostring method. (TIFF 2 MB)

## Abbreviations

AR: Adventitious root
MT: Microtubule
MAP MT: Associated protein
IBA: Indole butyric acid.
