# Supplementary figures and images for: Gene expression profiling in juvenile and mature cuttings of Eucalyptus grandis reveals the importance of microtubule remodeling during adventitious root formation
Source: BMC Genomics. 2014 Sep 30;15(1):826. doi: 10.1186/1471-2164-15-826 (PMC4190485; doi:10.1186/1471-2164-15-826)

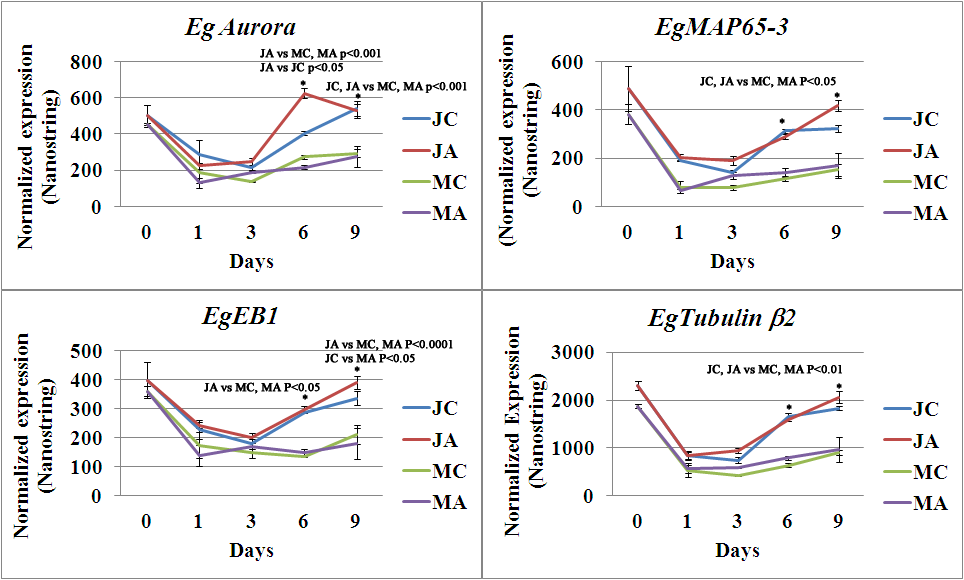

Supplement: Supplementary file 2 — Additional file 2: Figure S1: Scheffe analysis of the expression of four microtubule related transcripts. Chart represent averages and standard errors, and asteriks show statisticaly significant differences. Expression was determined by the nanostring method. (TIFF 2 MB) [file 12864_2014_6504_MOESM2_ESM.tiff]
